# Physiological Contribution in Spontaneous Oscillations: An Approximate Quality-Assurance Index for Resting-State fMRI Signals

**DOI:** 10.1371/journal.pone.0148393

**Published:** 2016-02-12

**Authors:** Ai-Ling Hsu, Kun-Hsien Chou, Yi-Ping Chao, Hsin-Ya Fan, Changwei W. Wu, Jyh-Horng Chen

**Affiliations:** 1 Institute of Biomedical Electronics and Bioinformatics, National Taiwan University, Taipei, Taiwan; 2 Brain Research Center, National Yang-Ming University, Taipei, Taiwan; 3 Graduate Institute of Medical Mechatronics, Chang Gung University, Taoyuan, Taiwan; 4 Institute of Neuroscience, National Yang-Ming University, Taipei, Taiwan; 5 Department of Biomedical Sciences and Engineering, National Central University, Taoyuan, Taiwan; 6 Brain and Consciousness Research Center, Taipei Medical University-Shuang Ho Hospital, New Taipei, Taiwan; National Yang-Ming University, TAIWAN

## Abstract

Resting-state fMRI (rs-fMRI) is receiving substantial attention for its sensitivity to functional abnormality in the brain networks of people with psychiatric and neurological disorders. However, because of the variety of rs-fMRI processing methods, the necessity of rs-fMRI quality assurance is increasing. Conventionally, the temporal signal-to-noise ratio (tSNR) is generally adopted for quality examination, but the tSNR does not guarantee reliable functional connectivity (FC) outcomes. Theoretically, intrinsic FC is supposed to reflect the spontaneous synchronization of neuronal basis, rather than that from thermal noise or non-neuronal physiological noise. Therefore, we proposed a new quality-assurance index for rs-fMRI to estimate the physiological contributions in spontaneous oscillations (PICSO). The PICSO index was designed as a voxel-wise measure for facilitating practical applications to all existing rs-fMRI data sets on the basis of two assumptions: Gaussian distributions in temporal fluctuations and ultra-slow changes of neural-based physiological fluctuations. To thoroughly validate the sensitivity of the proposed PICSO index to FC, we calibrated the preprocessing steps according to phantom data and verified the relationship between the PICSO and factors that are considered to affect FC in healthy participants (*n* = 12). Our results demonstrated that FC showed a significantly positive correlation with the PICSO. Moreover, for generating robust FC outcomes, directly acquiring data at a relatively large voxel size was more effective than performing smoothness on high-resolution data sets. In conclusion, compared with tSNR, the PICSO index is more sensitive to the resulting FC, providing a practical quality-assurance indicator for all existing rs-fMRI data sets.

## Introduction

The brain at rest is composed of multiple functional networks that have been extensively explored using connectivity approaches. Resting-state functional connectivity (rsFC) measures the synchronization of low-frequency blood-oxygen-level dependent (BOLD) oscillations [1], which are presumed to be the surrogate of spontaneous neuronal cross-talks [2–4]. Although scientists do not fully understand the intrinsic essence of resting-state fMRI (rs-fMRI) signals, numerous studies have demonstrated that rsFC can be altered by several neurological, psychiatric, and neurodegenerative diseases (e.g., Alzheimer's disease, Parkinson’s disease, depression, dementia, and schizophrenia) and can be dynamic among physiological conditions (e.g., anesthesia or sleep) [5–7]. A current trend in the proliferation of rs-fMRI investigations is to perform data mining from multi-center data sets for large samples (e.g., The Alzheimer’s Disease Neuroimaging Initiative, 1000 Functional Connectomes Project, and Human Connectome Project) [8]. However, because of the various types of data acquisition and experimental conditions, a promising quality-evaluation strategy is warranted for producing reliable rsFC outcomes.

Currently, a common measure of rs-fMRI data quality is the temporal signal-to-noise ratio (tSNR), which is the ratio of the mean signal over its temporal standard deviation (SD) [9–12]. Triantafyllou et al. tested the dependence of the tSNR on scanning parameters, such as magnetic field strength, flip angle (FA), image resolution, and echo time (TE) [9], and suggested the optimal conditions for enhancing the tSNR [13]. Although the tSNR provides an initial indication of rs-fMRI data quality, tSNR changes are not directly reflected in rsFC alterations [9,13,14]. In other words, a high tSNR does not guarantee reliable connectivity strength (CS). An extreme example of this is that the fMRI data from the phantom possess a high tSNR, but these data do not result in long-distance connectivity. This is because, by definition, the tSNR emphasizes the baseline average of rs-fMRI time courses. However, this baseline information does not contribute to rsFC; instead, the temporal fluctuations take the major contribution to rsFC outcomes. More specifically, the temporal fluctuations in rs-fMRI signals can be regarded as a combination of spontaneous neural activities, non-neuronal fluctuations (i.e., respiration and cardiac pulsation), and thermal noise from scanner electronics. Conceptually, rsFC results from the synchronization of spontaneous neural activities, whereas non-neuronal fluctuations and thermal noise are irrelevant to neuronal synchronization but are inevitably involved in rs-fMRI signals. Therefore, the physiological contributions in spontaneous oscillations, once quantified, should be a meaningful candidate for measuring the sensitivity of functional connectivity.

However, quantifying the physiological contributions in spontaneous activities is not a trivial task. Apart from hardware imperfections [15], the physiological contributions in rs-fMRI signals were first addressed by the Krüger physiological noise model [9,12]. This model assumes that the noise variance in the imaging voxels is composed of thermal noise, non-neuronal fluctuations, and spontaneous fluctuations of a potentially neuronal origin. Therefore, if the thermal noise could be estimated according to rs-fMRI signals, then the physiological contributions over thermal noise could be defined as a quality measure for rs-fMRI. This concept has previously been addressed, demonstrating the dependence of physiological contributions on the acquisition parameters. Triantafyllou et al. investigated the improvement of physiological contributions at a high field strength, large FA, and low spatial resolution [9]. Bodurka et al. suggested the optimal fMRI voxel size when the thermal noise matches the physiological fluctuations [10]. Additionally, Gonzalez-Castillo presented that the physiological contributions were more sensitive to the FA than the tSNR was [16]. Although these studies have emphasized the importance of physiological contributions for fMRI signals, their quantification strategies are time-consuming and unrealistic for application to existing rs-fMRI data sets and clinical routines.

Conceptually, a practical measure for quantifying physiological contributions should be voxel-wise, free from region of interest (ROI) selection, and without changing parameters in imaging acquisition. In Krüger’s model, the total fluctuation level is directly measured according to the SD of reconstructed fMRI images over time [12]; however, consensus on estimating thermal noise is difficult to achieve because this metric depends on how and where the thermal noise is defined. Two methods that are generally used for estimating thermal noise are measuring the spatial noise from a noise-only region or measuring the temporal noise without radio-frequency (RF) excitation. The first approach is more widely used than the second, but its applicability to rs-fMRI data is limited for three reasons: (a) manual selection of the noise region outside of the image object is required for calculating the background noise level; (b) regardless of imaging artifacts, hundreds of pixels are required in order to obtain a reasonable estimate of background noise because the precision of the noise estimation is proportional to the square root of the number of pixels in the ROI; (c) the suitability of manual ROI selection is questionable in parallel imaging because each channel contributes differently across the entire field of view. Compared with the first approach, the second approach without RF excitation is a more straightforward estimation method for assessing pure thermal noise over time. However, it requires hardware, pulse sequence editing, longer acquisition time, and a special reconstruction algorithm [17,18]. Although the second approach is robust and precise, it is still generally unfeasible as a practical estimation surrogate for existing rs-fMRI data sets.

To address these concerns, we propose a new voxel-wise method for estimating the PhysIological Contributions in Spontaneous Oscillations (PICSO) of the acquired rs-fMRI data sets. With this method, the thermal noise over time of the rs-fMRI signals is estimated in approximation by subtracting the imaging signals between each pair of adjacent time points; this is an extended version of the difference method [17,19]. This subtractive strategy enables high-frequency signals to be emphasized and low-frequency signals to be attenuated. In such an estimation, the physiological contributions are assumed to be negligible after the voxel-wise subtraction, and the resultant residues can be regarded as the source of thermal noise because the spontaneous rs-fMRI signals generally fluctuate at very low frequencies (<0.1 Hz), close to a time-invariant characteristic for every pair of adjacent time points. To validate the applicability of the proposed method in thermal noise estimation, we first verified that fMRI signals acquired from the phantom possessed a zero PICSO value after we performed the calibration procedure. Subsequently, we observed a positive relationship between the CS and PICSO at various image resolutions because the image resolution has been reported as the major factor affecting the CS [9,13,14]. Moreover, we conducted various degrees of spatial smoothing on the rs-fMRI data to confirm that the alteration in the CS can be directly reflected by the PICSO. This new approach produces several advantages for quantifying the physiological contributions in fMRI signals such as the applicability to parallel MRI [19] and to all rs-fMRI data sets for quality assurance.

## Material and Methods

### Theory

All rs-fMRI signals can be regarded as a superposition of the intrinsic baseline signal and signal fluctuations. The quality of rs-fMRI data is typically measured using the tSNR, which is defined as the ratio of the baseline average $\left(\bar{s}\right)$ to its SD over time (***σ***):

$$tSNR=\frac{\bar{s}}{\sigma }$$ (1)

Assuming that the rs-fMRI signals are free from non-neuronal fluctuations (i.e., respiration and cardiac pulsation), the signal variance can be separated into thermal noise and the desired physiological fluctuations of neural origin [20–23]. According to Krüger’s model, the total signal fluctuations in the fMRI signal (***σ***) are the square-law sum of the Gaussian thermal noise (***σ***_**0**_) and physiological fluctuations (***σ***_***p***_), expressed in equation form as $\sigma \;=\;\sqrt{\sigma_{0}^{2}+\sigma_{p}^{2}}$ [12], where ***σ***_**0**_ is independent of the fMRI signal intensity and ***σ***_**p**_ is scaled relative to the image intensity. Estimating the variance of both total fluctuation level and thermal noise facilitates calculating the ratio of physiological fluctuations to thermal noise, which is determined using Eq (2):

$$\frac{\sigma_{p}}{\sigma_{0}}=\sqrt{{\left(\frac{\sigma }{\sigma_{0}}\right)}^{2}-1}$$ (2)

where the $\frac{\sigma_{p}}{\sigma_{0}}$ ratio represents the fMRI PICSO and can be regarded as the sensitivity surrogate in the rs-fMRI signals. Additionally, for any non-ideal circumstance that causes the ratio of total fluctuation level over thermal noise to be less than unity, the PICSO value would be set to zero.

### Image Acquisition

A total of 12 right-handed healthy volunteers (age: 26.4 ± 2.1 y, females/males: 6/6) were enrolled in this study. All participants declared that they fully understood the experimental procedure and provided written informed consent. The entire procedure was approved by the Institutional Review Board of National Yang-Ming University. Data were acquired using a Siemens 3T Trio system with a 12-channel head coil. To verify the accuracy of the thermal noise estimation in the PICSO, a spherical water phantom consisting of 1.25 g of NiSO_4_·6H_2_O per 1000 g of distilled water was scanned using identical imaging protocols. T_1_-weighted structural images were obtained using the MP-RAGE sequence (TE = 2.27 ms, repetition time [TR] = 1.9 s, inversion time [TI] = 900 ms, FA = 9°, 176 slices with 1 × 1 × 1 mm^3^ voxels without an interslice gap). Given the fact that the weightings of thermal noise deviate with voxel sizes [14], we acquired the rs-fMRI signals at various image resolutions as the dominant factor to manipulate the PICSO values for each single subject. Thereafter, the single-shot gradient-echo echo planar imaging (GE-EPI) sequence was adopted to acquire rs-fMRI data at four voxel sizes (1.3 × 1.3 × 2, 2 × 2 × 2, 3 × 3 × 3, and 5 × 5 × 5 mm^3^) by using the parameters shown in Table 1. Each session contained 150 time points and three dummy scans with a total acquisition time of 7 min 38 s. The scanning order of the four EPI sessions was counterbalanced in a Latin-square manner to reduce the systematic bias resulting from the scanning order of the EPI sessions. Because of the limited brain coverage for sessions at the highest spatial resolution (1.3 × 1.3 × 2 and 2 × 2 × 2 mm^3^), we assigned a slice orientation along the anterior and posterior commissure lines with the midline of the slab reaching the bottom edge of the corpus callosum to cover the thalamus (THAL) and posterior cingulate cortex (PCC). For the other sessions, we maintained the same slice orientation and ensured the coverage of the entire brain. The participants’ heads were immobilized using cushions to minimize motion during image acquisition. During the rs-fMRI sessions, the participants were instructed to open their eyes, relax, and not think of anything specific. To minimize the contributions of non-neuronal sources embedded in the rs-fMRI signals, simultaneous cardiac and respiratory recordings were acquired using a built-in pulse oximeter and pneumatic belt, respectively (sampling rate = 50 Hz). A B_0_ field map was acquired using a dual-echo gradient echo sequence (TE_1_ = 10 ms, TE_2_ = 12.46 ms, TR = 600 ms, FA = 70°, 33 slices with 1.5 × 1.5 × 4 mm^3^ voxels) to correct image distortions caused by the inhomogeneity of B_0_. The total acquisition time was 38 min 38 s.

**Table 1:** Acquisition parameters for the EPI with four spatial resolutions.

Acquired Voxel Size (mm^3^)	FOV (mm)	Matrix Size
1.3 × 1.3 × 2.0	162 × 162	128 × 128 × 27
2.0 × 2.0 × 2.0	256 × 256	128 × 128 × 27
3.0 × 3.0 × 3.0	192 × 192	64 × 64 × 35
5.0 × 5.0 × 5.0	320 × 320	64 × 64 × 24

TR = 3000 ms, TE = 35 ms, flip angle = 87°,

Partial Fourier = 6/8, bandwidth = 1260 Hz/px, Echo spacing = 0.86 ms.

### fMRI Preprocessing

**Fig 1:**
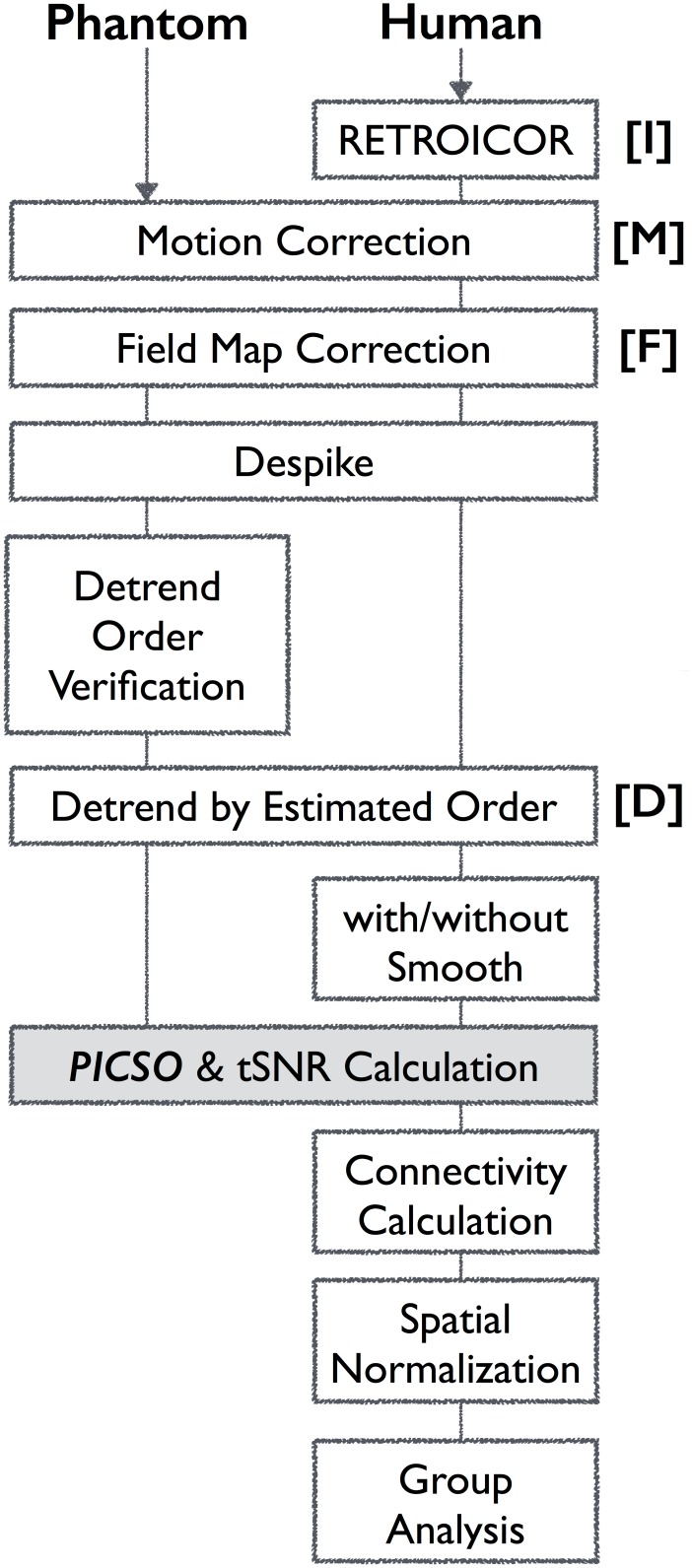
Flowchart of the rs-fMRI preprocessing procedure and PICSO estimation approach.

All scanning images were preprocessed using AFNI [24] and FSL [25]. During preprocessing, spatial smoothing is a crucial factor that affects the tSNR [13] and CS [26]; thus, to prevent the additional smoothing induced by spatial normalization, all functional images were analyzed in the native space, including both preprocessing and the seed-correlation analysis, and finally transformed into the MNI space for group analysis. Fig 1 demonstrates the workflow of phantom—human preprocessing. The phantom data set first underwent standard preprocessing (Fig 1) including motion correction, field map correction, and despiking. Subsequently, different detrending orders were performed to verify the signal drift induced by system instability. The detrending order was then determined when the thermal noise was equal to the total fluctuation level because the phantom possessed the stationary baseline signal and lacked physiological fluctuations. For the human data, the effects of cyclic cardiac pulsation and respiration were first removed using RETROICOR with second-order Fourier series expansion (3dretroicor in AFNI) [20]. Motion correction was then performed using FSL (mcflirt) to minimize possible head movement for each rs-fMRI time series. Retrospective field-map correction was then conducted using FSL (fugue) to eliminate the image distortions caused by the field inhomogeneity. The spike (3dDespike in AFNI) and estimated polynomial trends were removed from the time series. Moreover, because head motion at both the individual and group levels can contribute to spurious correlations [27,28], we examined the motion by using mean framewise displacement (FD) for the entire rs-fMRI data set. All rs-fMRI data fulfilled the motion criteria (i.e., mean FD < 0.3 mm). To further examine the effects of head motion on the PICSO, we performed a correlation analysis between the PICSO and mean FD for comparison.

### Estimating the Thermal Noise and PICSO

After the preprocessing, for a given voxel, the tSNR was determined using Eq (1) according to the ratio of the mean signal intensity **$\bar{s}$** to the SD ***σ*** of a series of 150 functional images. Because the rs-fMRI signals were modeled as the sum of true BOLD intensity and the superimposed temporal noise, which following a Gaussian distribution [29], the slow fluctuations in rs-fMRI in adjacent acquisitions were assumed to be canceled out by subtraction, resulting in Gaussian-noise residue. Therefore, the voxel-wise thermal noise ***σ***_**0**_ was determined by calculating the SD of a series of subtractive images between adjacent scans and divided by $\sqrt{2}$ because the SD of the subtracted Gaussian noise is theoretically increased by $\sqrt{2}$ [19]. After the maps of thermal noise ***σ***_**0**_ and total noise ***σ*** were calculated, the PICSO map was determined as the ratio of the variance of the total fluctuation level to the thermal noise, as shown in Eq (2). Subsequently, the tSNR and PICSO values were averaged within the predefined ROIs for a given data set at various spatial resolutions. For the phantom results, we expected the thermal noise to be identical to the total fluctuation level, and the PICSO index to be zero.

### Seed-Based Correlation Analysis for Functional Connectivity

We conducted a seed-based correlation analysis by using the THAL to represent the subcortical structures and the PCC to signify the cortex representative. According to the data sets of four resolutions, the seed regions were identified in the native space through the following three steps: (1) The seed points of the left THAL and PCC were first defined using the MNI coordinates (−7, −16, 6) and (2, −51, 27), respectively [30,31]. (2) These seed points were inversely transformed from the MNI space back to the reference data set (3 × 3 × 3 mm^3^) in the native space, and the native seed regions were then prescribed a sphere with a 5-mm radius on the reference data set. (3) The native seed regions of the other resolutions (i.e., 1.3 × 1.3 × 2, 2 × 2 × 2, and 5 × 5 × 5 mm^3^) were transformed from the native seed region on the reference data set to match the image resolutions by using FSL (flirt). Following these steps ensured that the native seed regions were identical among the various imaging sessions to prevent the bias of seed size and the resulting CS.

Prior to FC calculation, we conducted nuisance regression by using the following 10 covariables: six affine motion parameters, two temporal variations of the respiration volume and heart rate [22], and the mean time series of white matter (WM) and cerebrospinal fluid (CSF). The WM and CSF masks were generated from the segmented T_1_ anatomical image by using FSL fast with a threshold 50% probability, and transformed to match the image resolutions. Subsequently, the FC maps were obtained through a seed-regression analysis in the native space by extracting the average residual time series from the resolution-matched seed regions and using it as the regressor against every voxel in the brain by AFNI (3dDeconvolve). The correlation maps from the regression model were then converted to Fisher’s z maps. For each seed, the same procedures for seed prescription and seed correlation were applied to the four image resolutions and four smoothing levels, resulting in 16 FC maps for each participant. Setting the smoothing levels lower than the acquired image resolution did not affect the data sets; therefore, we report only 10 FC maps corresponding to the effective changes in spatial resolution. For the group analysis, the resulting z maps were normalized to the MNI space for a one-sample *t* test (3dttest++); the significance level was set to FDR-corrected p < 0.01 (3dFDR) with an explicit common mask among the participants. The normalization process is detailed in the following subsection.

### Normalization after Functional Connectivity

To avoid imposing extra smoothing effects on PICSO estimations, we only applied spatial normalization for visualizing group-level index maps. The first volume of the functional data sets in the four acquired voxel sizes was used as the reference scan to estimate the transformation matrix. Spatial normalization was applied to transform the FC maps in the native space to the MNI space by using the predefined transformation matrix. The predefined transformation matrix that was used to transform the FC maps from the native space to the MNI space was produced using a two-stage (the reference scan with 3 × 3 × 3 mm^3^ spatial resolution) or three-stage process (the scans with other spatial resolutions). For the reference data set with an acquired voxel size of 3 × 3 × 3 mm^3^, the reference scan was aligned with the T_1_ image (boundary-based registration). This aligned image was then nonlinearly warped to the MNI space by using the warping matrix, which was determined by warping the native T1 image to the MNI T1 image (fnirt). Next, the transformation matrices from the previous two stages were combined into a two-stage reference scan matrix to minimize the interpolation effect during spatial normalization. The other functional scans with the acquired voxel size beyond 3 × 3 × 3 mm^3^ were registered to the reference scan (flirt) prior to the two-stage process to produce a three-stage transformation matrix. Finally, all the FC maps were warped to the MNI space with a spatial resolution of 2 × 2 × 2 mm^3^ by using the two- or three-stage transformation matrices.

### Resolution and Spatial Smoothing

To investigate whether the CS alterations could be directly reflected by the quality measurements (tSNR and PICSO), we manipulated multiple noise levels by processing the data sets with various degrees of smoothness. The smoothness of each preprocessed fMRI data set was controlled by applying a smoothing kernel until the predefined uniform full-width-at-half-maximum (FWHM) (measured in millimeters) was reached, matching the spatial resolutions of the acquired images (i.e., 2 × 2 × 2, 3 × 3 × 3, and 5 × 5 × 5 mm^3^). Notably, the FWHM is the inverse of the shortest distance that discriminates two points, and is determined according to the acquired voxel size and applied smoothing level. We did not apply the conventional smoothing method with a fixed Gaussian kernel because of the uncontrolled FWHMs; instead, we expanded the point spread function to specific levels to compensate for the intrinsic point spread functions of image acquisition, which might have differed among the participants.

### ROI Analysis

To investigate whether the effect of the spatial resolution on the CS was consistent with that on both quality measurements (tSNR, and PICSO), Pearson’s correlation analysis was conducted among the CS, tSNR, and PICSO in both the cortical and subcortical areas. The left THAL (L THAL) and PCC were chosen for studying the local rsFC within the thalamic network and default mode network (DMN), and the right THAL and medial prefrontal cortex (MPFC) were set as proxies for examining the strength of distant rsFC within these networks. The selected ROIs of the thalamic network were obtained from the MNI template embedded in FSL and those of the DMN were obtained from a previous study [32]. All ROIs defined in the MNI space were warped to the native space corresponding to each acquired voxel size through inverse spatial normalization. The ROI analysis was performed to quantify the mean PICSO index, tSNR values, and CS in the native space. In addition, at the same FWHM, repeated-measures analysis of variance (ANOVA) was employed to compare the averaged PICSO values among the data sets in various voxel sizes, and Pearson’s correlation analysis was used to examine the relationship between the quality measurements (tSNR and PICSO) and CS. Additionally, to further examine whether RETROICOR and imaging resolution affected the noise estimation, the average of both ***σ***_**0**_ and ***σ*** within the predefined ROIs was examined using two-way repeated-measures ANOVA (a 2 × 4 design, Factor 1: with and without applying RETROICOR, Factor 2: the four acquired imaging resolutions).

## Results

### PICSO Calibration and Estimation

**Fig 2:**
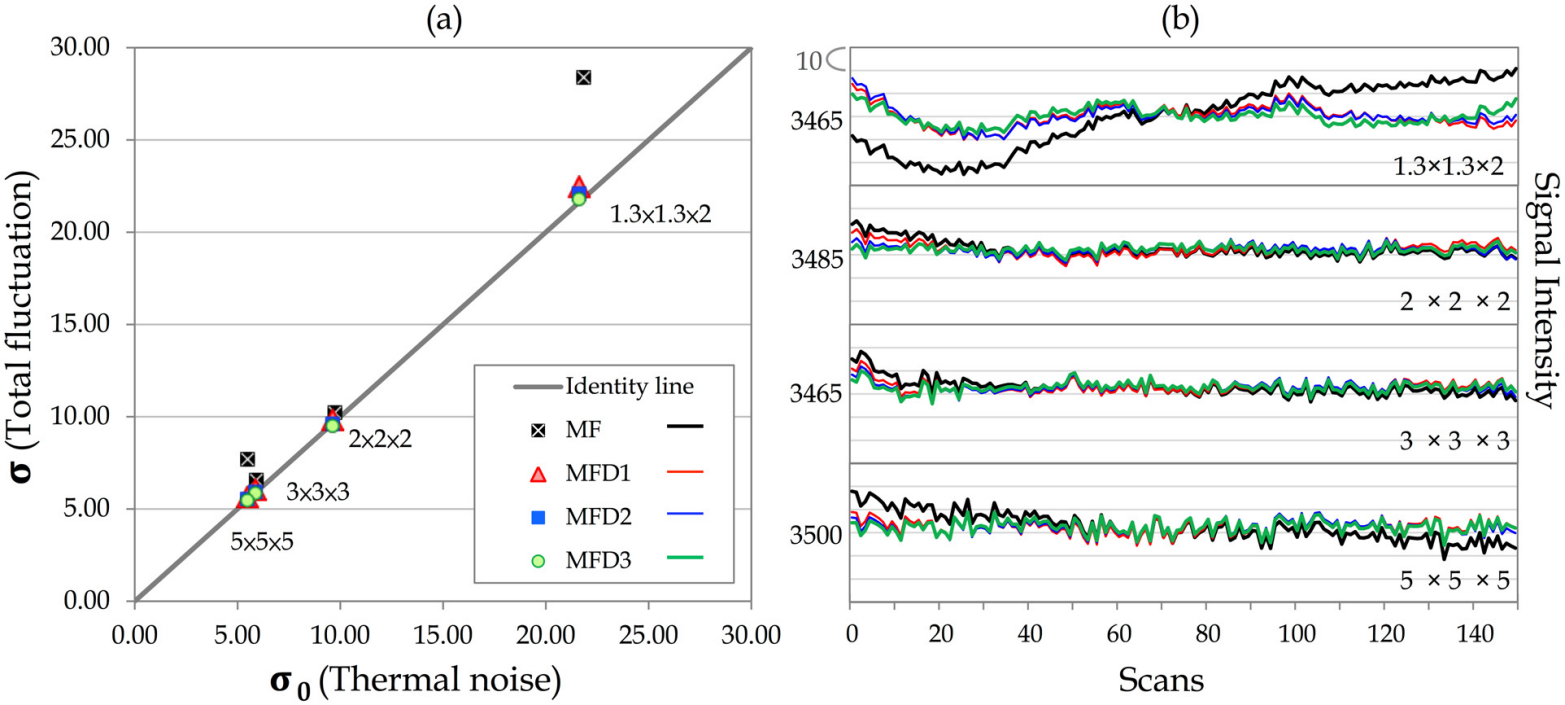
PICSO calibration using phantom data. (a) Total fluctuation level and the estimated thermal noise depend on the acquired spatial resolution and the preprocessing steps. The solid line represents the identity line (i.e., PICSO = 0). The solid black rectangle represents the results processed by motion correction (M) and field-map correction (F). Results with different levels of detrending (D) orders are denoted as D1, D2, and D3. The thermal noise and total fluctuation level showed identical changes when the detrending order was set as a cubic polynomial. (b) Corresponding time series without detrending and with different detrending orders among the four acquired voxel sizes. Time series processed without detrending and processed with the first, second, and third detrending orders are represented by the black, red, blue, and green lines, respectively. The numbers that appear on the left side of the time series represent the average signal intensity. Each line space indicates an intensity increment of 10.

Phantom data were used in the first step of calibration; Fig 2a depicts the total fluctuation level as a function of thermal noise under different voxel sizes of acquisition (1.3 × 1.3 × 2, 2 × 2 × 2, 3 × 3 × 3, and 5 × 5 × 5 mm^3^) and various detrending orders in preprocessing. The total fluctuation level decreased as the detrending order was set from linear, quadratic to cubic polynomial, whereas the thermal noise was free from the effect of detrending, indicating that signal trends contributed dominantly to the estimation of total fluctuation level. The influence of detrending on the fMRI time series is shown in Fig 2b for different acquired voxel sizes. With the detrending order as cubic polynomial, the scatter points in Fig 2a are located on the identity line (PICSO = 0) among different acquired resolutions, verifying the reliability of thermal noise estimation on the basis of the subtracting procedure [18,19,33].

Using the human data set as the second step of calibration, we attempted to validate that ***σ*** is sensitive to physiological noise, but ***σ***_**0**_ is not. We conducted a repeated-measure two-way ANOVA to test the RETROICOR effect on both thermal noise and total fluctuation level within the predefined seed-side ROI. For the L THAL, the ***σ*** estimation of data processed with RETROICOR differed significantly from that processed without RETROICOR (F(1, 10) = 30.12, p < 0.05), whereas the RETROICOR process did not significantly affect the estimation of ***σ***_**0**_ (F(1, 10) = 0.87, p = 0.37). In the PCC, the ***σ*** estimation of data processed with RETROICOR differed significantly from that processed without RETROICOR (F(1, 10) = 32.34, p < 0.05), whereas the data processed with RETROICOR did not significantly affect the estimation of ***σ***_**0**_ (F(1, 10) = 0.02, p = 0.88). For testing this concept throughout the brain, we adopted the same test for multiple ROIs in the MNI template embedded in FSL (Harvard—Oxford Subcortical Structural Atlas). Because of the limited spatial coverage of the acquired images, the ROIs could be used for testing only the bilateral cerebral cortex, THAL, caudate, putamen, and pallidum. The results are included in this manuscript as supplementary information (S1 Table). The observations indicated that the ***σ***_**0**_ estimation is appropriate for estimating the noise, which is irrelevant to physiology. Considering the influence of head motion on the PICSO estimation, we performed a correlation analysis to test the association between the PICSO values in the predefined ROIs and mean FD. No significant correlations were detected between the mean FD and PICSO for any ROIs, including the bilateral THAL, PCC, and MPFC (p > 0.35).

**Fig 3:**
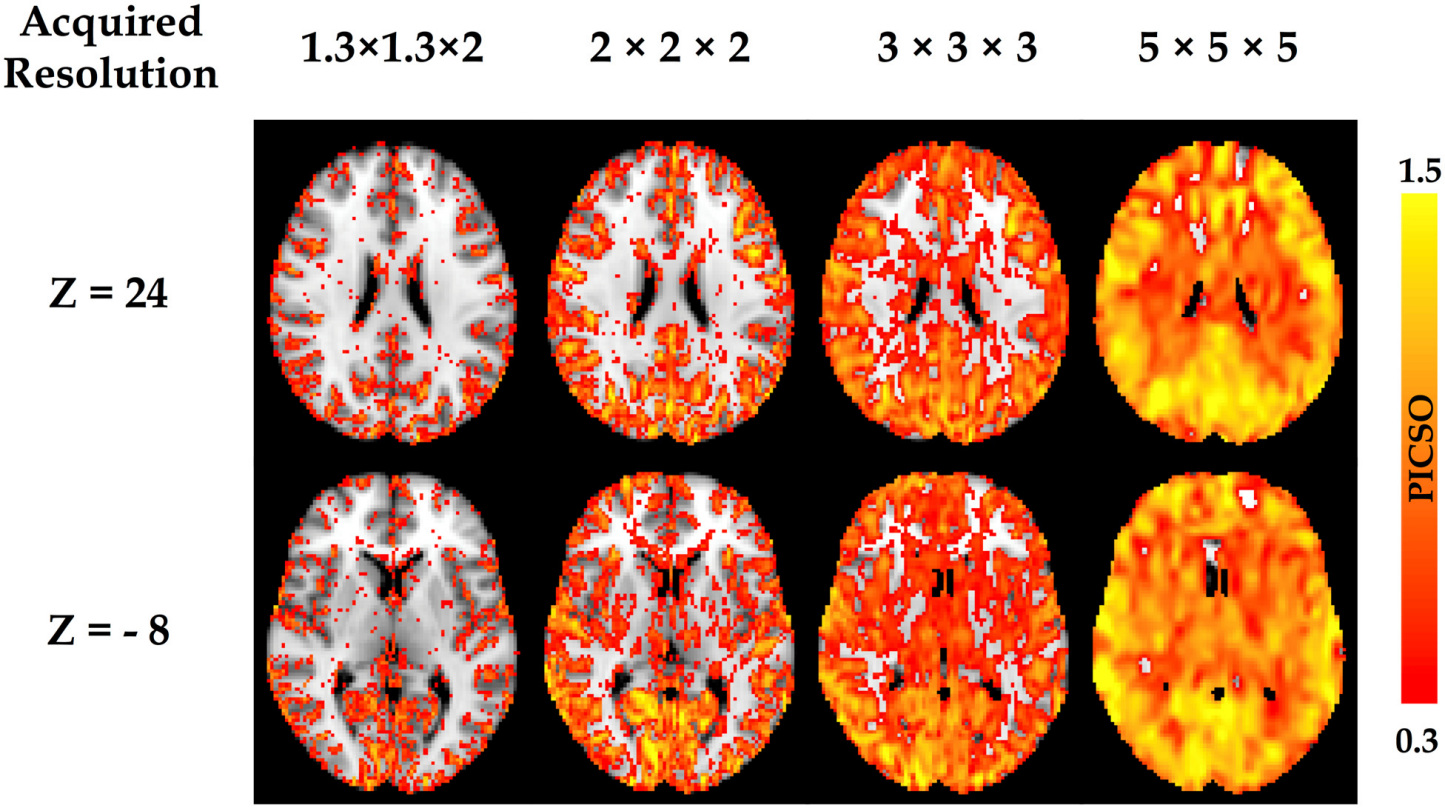
Voxel-wise PICSO map from a single subject. The PICSO value increases as a function of the acquired voxel sizes (1.3 × 1.3 × 2, 2 × 2 × 2, 3 × 3 × 3, and 5 × 5 × 5 mm^3^, without smoothing). Additionally, the PICSO value also possesses spatial specificity; the PICSO value of the posterior brain is generally larger than that of the anterior brain.

Fig 3 shows the PICSO map from a single subject as a function of the acquired voxel sizes (1.3 × 1.3 × 2, 2 × 2 × 2, 3 × 3 × 3, and 5 × 5 × 5 mm^3^, without smoothing). The PICSO value increased with the acquired voxel size and presented the spatial specificity. The PICSO value in the neocortex was higher than that in the subcortical region. For all cortical regions, the PICSO value in the posterior brain was higher than that in the anterior brain, suggesting relatively high physiological contributions in the posterior brain.

### PICSO Modulated by the Acquired Voxel Size

**Fig 4:**
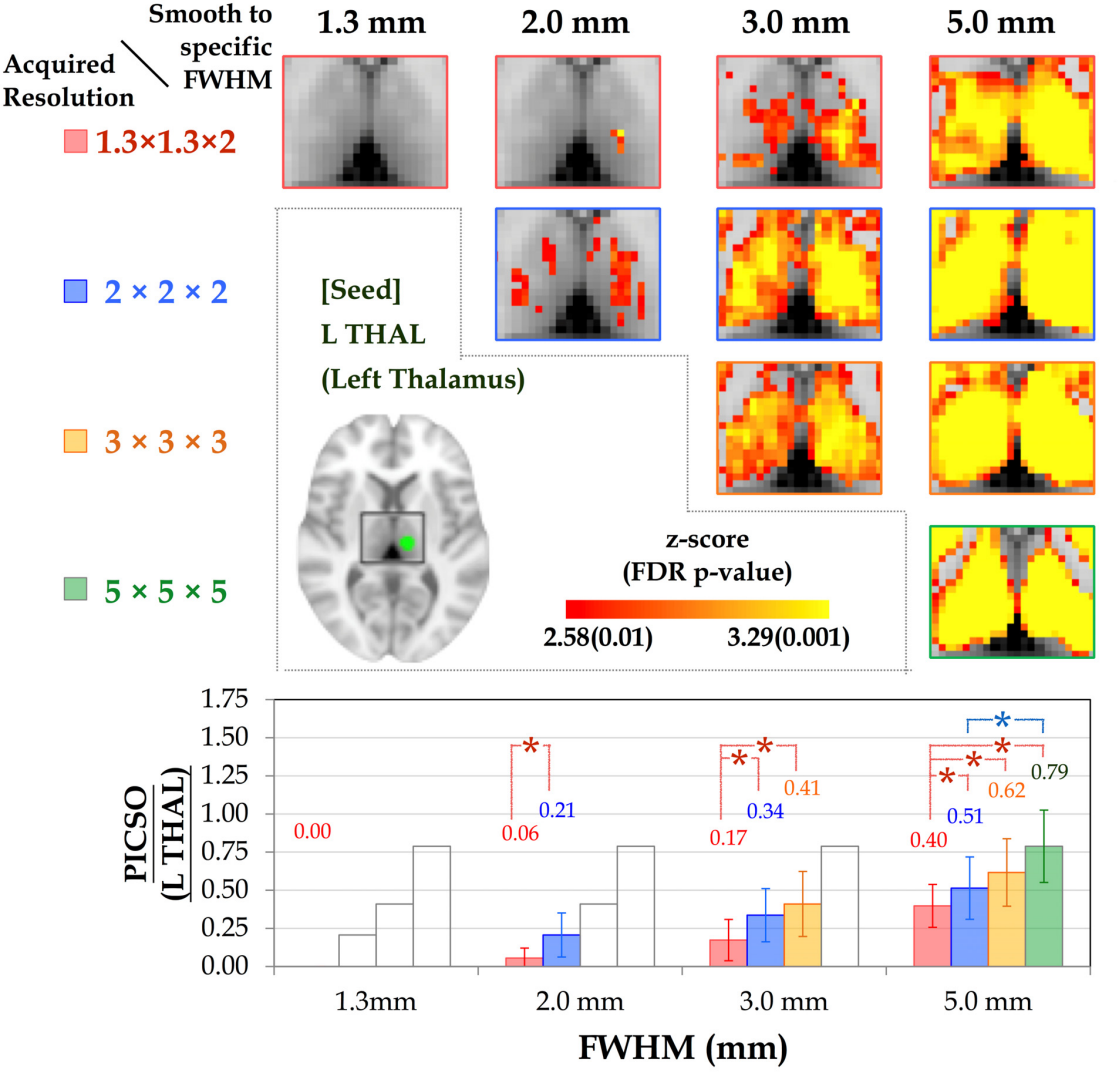
Group-level thalamic connectivity as a function of spatial resolution. (Upper) The thalamic connectivity varies with smoothness (FWHMs = 1.3, 2, 3, and 5 mm) under the four acquired voxel sizes. Although the spatial extent of FC is preserved among the various voxel sizes, the CS increases with the smoothness. (Lower) Bar chart illustrating the corresponding PICSO value for each smoothness level of the acquired voxel sizes. Both the red and blue lines indicate significant differences in the paired comparisons (LSD-corrected p < 0.01).

**Fig 5:**
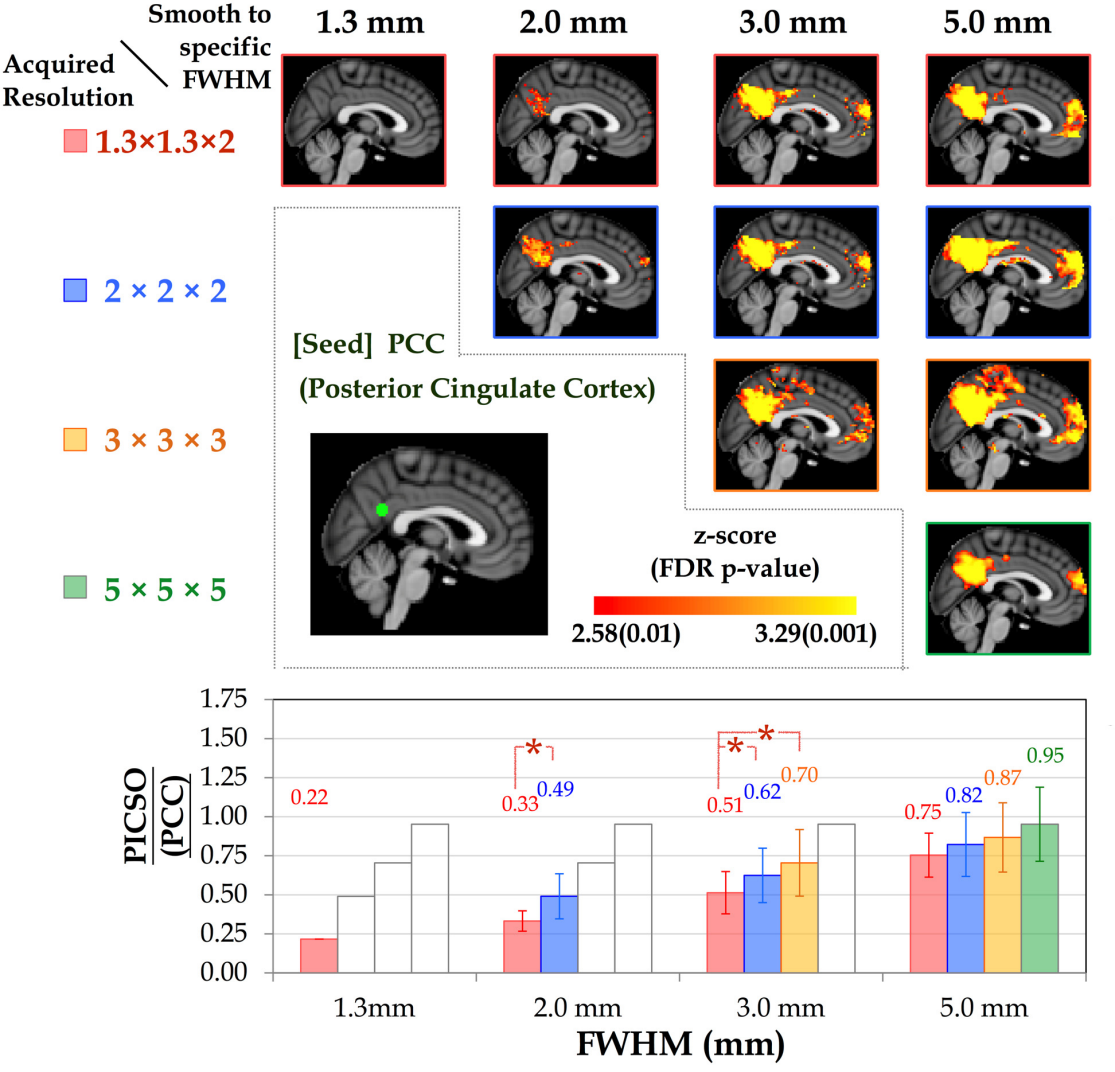
Group-level DMN connectivity as a function of spatial resolution. (Upper) DMN connectivity varies with smoothness (FWHMs = 1.3, 2, 3, and 5 mm) under the four acquired voxel sizes. (Lower) Bar chart illustrating the corresponding PICSO values for each smoothness level of the acquired voxel sizes. Both the red and blue lines indicate significant differences in the paired comparisons (LSD-corrected p < 0.01).

The diagonal pictures of the upper panels in Figs 4 and 5 depict the FC as a function of the acquired voxel sizes in the thalamic network and DMN, respectively. In both networks, the mean CS increased monotonically with the voxel size (p < 0.05). As the voxel size increased, the mean CS (±SD) in the L THAL increased from 0.06 (±0.03) to 0.16 (±0.09), 0.28(±0.10), and 0.40 (±0.09), and the corresponding PICSO increased from 0.00 (±0.00) to 0.21 (±0.14), 0.41 (±0.21), and 0.79 (±0.24). In the PCC, as the voxel size increased, the mean CS increased from 0.09 (±0.01) to 0.19 (±0.01), 0.27 (±0.02), and 0.32 (±0.03), and the corresponding PICSO increased from 0.22 (±011) to 0.49 (±0.15), 0.70 (±0.20), and 0.95 (±0.22).

### PICSO Modulated by the Smoothness

The off-diagonal pictures in the upper panels of Figs 4 and 5 show the smoothing effects of the FC maps on the acquired voxel sizes in the thalamic network and DMN, respectively. For any fixed voxel size in acquisition, spatial smoothing substantially enhanced the CS and spatial extent within the network. However, when the FWHM was controlled after smoothing, the impact of the acquired voxel size on the CS was more substantial than the impact of smoothing. For example, the CS of the data acquired at 2 × 2 × 2 mm^3^ was significantly stronger than that of the data acquired at 1.3 × 1.3 × 2 mm^3^ and smoothed to the uniform FWHM of 2 × 2 × 2 mm^3^ (FDR-corrected p < 0.01). The lower panels of Figs 4 and 5 show the relationships between the PICSO values and the fixed FWHMs after smoothing; the bars represent the multiple voxel sizes in acquisition. The transparent bars indicate incidences where the expected FWHMs after smoothing were less than the acquired spatial resolution, indicating that the smoothness had no effect. For the fixed FWHM, both the red and blue lines indicate a significant difference among acquired voxel sizes at the significance level of a Fisher’s least significant difference (LSD)-corrected p < 0.01. For example, for the final FWHM of 3 mm in the lower panel of Fig 4, the data with acquired voxel sizes of 2 × 2 × 2 and 3 × 3 × 3 mm^3^ had higher PICSO values than those with an acquired voxel size of 1.3 × 1.3 × 2 mm did, and no significant difference in the PICSO values was observed between the data with acquired voxel sizes of 2 × 2 × 2 and 3 × 3 × 3 mm.

### Relationship between the PICSO and CS

**Fig 6:**
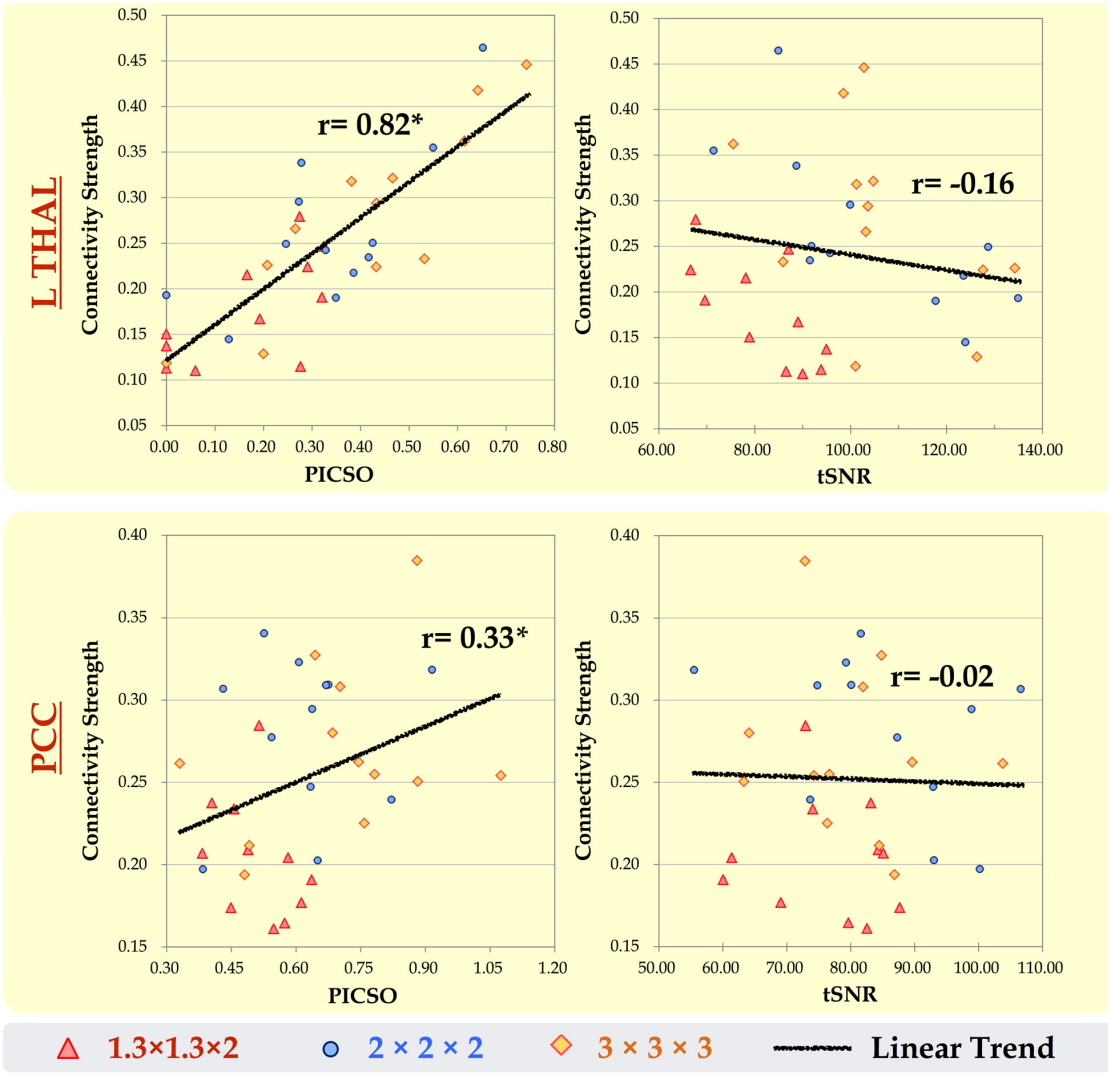
Relationship between both quality measurements (PICSO and tSNR) and the CS in the rs-fMRI data sets within the L THAL and PCC. At a fixed FWHM of 3 mm, each point in the scatter plot indicates an individual subject, and the linear trend line denotes the coupling among the three data sets of the acquired voxel sizes of 1.3 × 1.3 × 2, 2 × 2 × 2, and 3 × 3 × 3 mm^3^. Only the trend lines between the PICSO and CS correlate significantly within the L THAL (r = 0.82) and PCC (r = 0.33).

To verify the efficacy of using the PICSO or tSNR as quality measures for rs-fMRI, we conducted a correlation analysis to quantify the association between the CS and the two quality measurements after we controlled the smoothness. According to the data of three voxel sizes (1.3 × 1.3 × 2, 2 × 2 × 2, and 3 × 3 × 3 mm^3^, which are conventionally adopted in human studies, all of which were smoothed to a fixed FWHM of 3 mm), Fig 6 shows the relationship between the CS and both quality measurements (PICSO and tSNR) within the predefined seed ROIs. Each point in Fig 6 indicates an individual subject, and the trend lines denote the coupling between the quality measurements and the CS among the three data sets. The PICSO and CS demonstrated a large significantly positive correlation within the L THAL (r = 0.82, p < 0.05) and a medium significantly positive correlation with the PCC (r = 0.33, p < 0.05). However, the tSNR, which is commonly adopted to test fMRI quality, showed a nonsignificant correlation with the CS. These phenomena indicated the high sensitivity of the PICSO index to the FC in the rs-fMRI signals.

## Discussion

Recently, data quality in rs-fMRI has received substantial attention because different imaging centers often adopt different rs-fMRI protocols. In principle, tSNR could be used as a quick measure of rs-fMRI data quality, but it does not guarantee reliable rsFC outcomes. Previous studies have emphasized the temporal fluctuations in fMRI signals and the potential of physiological contributions to serve as a sensitivity indicator of rs-fMRI quality [9]. Nevertheless, current quality-evaluation strategies that involve manual ROI selection for determining physiological contributions are time-consuming and unrealistic when processing rs-fMRI data sets derived from a large sample [9,13,14]. Because of practical concerns, we first proposed a novel approach for estimating the physiological contributions in rs-fMRI signals under the assumption that the ultra-slow trends and low-frequency neuronal fluctuations can be minimized using temporal subtraction. We titled this estimation index as the PICSO for rs-fMRI, similar to the notion of the contrast-to-noise ratio in the stimuli-evoked fMRI signals. Crucially, the reliability of the proposed approach was verified through the phantom data with four frequently adopted voxel sizes (Fig 2), and the PICSO index was shown to be more sensitive to the resulting rsFC than the tSNR was. Second, our analysis of the various spatial resolution revealed that reducing the acquired image resolution or adopting a large smooth kernel increased the rsFC and physiological contributions, which is consistent with previous reports [9,13,14]. In brief, we achieved the following three aims of this study: (1) calibrating the PICSO estimation procedures by using a phantom, (2) verifying the high sensitivity of PICSO for detecting the CS (Fig 6), and (3) determining that the acquired voxel size had a larger effect on the PICSO than the smoothness did (lower panels in Figs 4 and 5). Moreover, the PICSO index has the advantages of avoiding additional acquisition, facilitating a practical quality evaluation for existing fMRI databases derived from a large sample, and enhancing the rs-fMRI reliability of future clinical investigations. Notably, however, the PICSO represent the weighting of physiological fluctuations over the thermal noise of a single voxel, whereas the CS is the temporal correlation between two voxels or regions. They differ conceptually, and their relationship is described as follows. High CS outcomes in regions must result from the sufficient PICSO (high physiological contribution) values in these regions; however, regions with high PICSO values do not directly indicate their FC. Accordingly, the linear correlation between the CS and PICSO (Fig 6) was medium to large when different noise levels were modulated (by imaging resolution or spatial smoothing), but in actuality (with a fixed noise level), the PICSO can only be regarded as the prior reliability measure for the rsFC.

According to the physiological noise model in BOLD-fMRI [9,12], the PICSO index was proposed to approximately estimate the contribution of intrinsic spontaneous activities by estimating the ratio of ***σ***_***p***_ over ***σ***_**0**_ in a voxel-wise manner without additional sequence editing. Between these two parameters, estimating ***σ***_**0**_ is more uncertain and difficult due to the existence of multiple approaches for doing so. The approach of estimating ***σ***_**0**_ without RF excitation has recently been well adopted because it is a straightforward concept, but its requirements of extra sequence editing and image reconstruction greatly lengthen the time required for experiments and analysis, rendering it almost impossible to apply to existing RS-fMRI data sets [17,18]. For image processing, a general method for estimating $\sigma_{0}$ is to calculate the spatial variance within a selected ROI outside of the brain region, and a subtractive imaging method was employed to subtract images from the selected two adjacent images and estimate the thermal noise in an ROI manner [19]. However, these ROI-based methods are not designed for generating a voxel-wise map and lack temporal information for rsFC. By contrast, we used a series of subtractive images from every two adjacent time points to enable accurate measurement of local ***σ***_**0**_, regardless of the image spatial noise, and to enable the practical application of the proposed method to existing rs-fMRI data sets. This subtractive strategy was based on two assumptions: (1) the temporal thermal noise follows a Gaussian distribution [29], and (2) the resting-state spontaneous activity does not change quickly for each pair of adjacent time point. If either assumption is violated by the rapid signal changes because of a subject’s head motion, then the robustness and accuracy of the PICSO estimation might decrease. Thus, for preprocessing, the rs-fMRI data were processed using motion correction and despiking to minimize the motion impact prior to the PICSO calculation. Although no existing motion correction methods could guarantee that the data were completely free from motion, the residual motion effect in our approach would be considered as the thermal noise because of the high-frequency enhancement by the subtractive strategy, thus reducing the PICSO value. Under the general inclusion criteria with the mean FD of less than 0.3 in our data set, the PICSO value showed no correlation with the mean FD, suggesting that head motion does not affect the PICSO.

Moreover, any slight signal drift during image acquisition also affects the PICSO estimation; hence, this drift should be corrected using the detrending procedure, the order of which might differ among scanners. In our preprocessing, the detrending order was set to the third order on the basis of the phantom verification because, in the phantom data, when the total fluctuation level is equal to the thermal noise; this indicates no physiological fluctuation contributions. Our phantom results (Fig 2a, with various acquired voxel sizes) verified the assumptions in the plot of ***σ*** versus ***σ***_**0**_ that followed the identity line after detrending, and the thermal noise determined by the proposed approach showed high stability under various acquired resolutions, demonstrating the reliability of the subtractive procedure [18,19,33]. Moreover, Fig 6 illustrates that the PICSO and CS were positively correlated, but the tSNR and CS did not show a significant correlation. Therefore, according to the two assumptions, PICSO estimation can be a convenient quality-assurance measure in the preprocessing procedure for rs-fMRI data sets. Notably, a higher PICSO value was displayed in the visual cortex compared with that in the THAL (Fig 3), which is consistent with the findings of Bianciardi et al. (2009). They also reported that the visual cortex showed higher sensitivity to the spontaneous activity than the entire gray matter did [23]. Because the rs-fMRI signals are based on the BOLD mechanism, the PICSO inhomogeneity among the brain regions implied a regional disparity in the cerebrovascular structure.

The off-diagonal upper panels in Figs 4 and 5 reveal the substantial influence of the spatial resolution on the rsFC, showing that the PICSO (lower panels in Figs 4 and 5) is an effective index for reflecting the FC changes. The results indicate that the brain areas with low PICSO values were confounded by thermal noise, causing a low CS and reducing the spatial extent. This may explain why studies have seldom reported high-resolution rsFC results without spatial smoothing. Although the relationship between the PICSO and CS was explicit, the PICSO values were lower than anticipated (approximately 0.00–0.95). To ascertain the confidence level, as a reference in the PICSO estimation, we examined the data sets of Bianciardi, who estimated the contributions of various noise sources in rs-fMRI data by using regression approaches [23]. After the signal drift and nonneuronal fluctuations were removed, the fMRI variance of Bianciardi’s 7T data sets resulted in a PICSO value of 0.6 in the whole brain gray matter and 1.7 in the visual cortex, indicating the spatial specificity of the PICSO value among brain regions. Similarly, according to their image resolution (1.25 × 1.25 × 2 mm^3^), the PICSO in the visual cortex was approximately 0.8 in the visual cortex of our high-resolution 3T data set (1.27 × 1.27 × 2 mm^3^). We concluded that this PICSO value was reasonable because the PICSO index is linearly proportional to the field strength, which is consistent with Huettle’s statement that “while thermal noise increases linearly with increasing field strength, physiological noise increases quadratically with the field strength” [34]. In current hardware settings, although the PICSO value in subcortical regions is low compared with the thermal noise level, this difficulty can be alleviated through spatial smoothing (Fig 4) or elevating the field strength.

Traditional spatial smoothing in fMRI preprocessing involves applying a smooth kernel with a fixed kernel size rather than smoothing until a uniform point-spread-function is reached, a procedure that was performed in this study. However, uniform smoothing was necessary in the current study to control the effective spatial resolution (i.e., the FWHM level) and to compare the PICSO and CS with the same criteria. Moreover, the artificial connectivity induced by head motion has attracted global attention in the rs-fMRI field [35]; applying uniform smoothness facilitates minimizing the motion-induced variability among participants [36]. The intrinsic blurring factors related to motion were minimized because the uniform smoothing performed using 3dBlurToFWHM entailed employing an iterative estimation scheme until an approximation of the desired smoothness was reached.

The PICSO value was inversely related to the effective spatial resolution and was affected by the acquired voxel size and the applied spatial smoothing. We defined the spatial resolution of the acquired voxel size (SRv) according to the intrinsic FWHM before smoothing, whereas the spatial resolution of smoothing (SRs) was associated with the final elevated FWHM (after the smoothing was executed) to a predefined level, regardless of high-resolution acquisition. Comparing the two factors at the same effective spatial resolution revealed that the SRv reduced ***σ***_**0**_ substantially more than the SRs did. In addition, increasing the voxel size increased the ***σ*/*σ***_**0**_ ratio, thus enhancing the PICSO value. At the same FWHM, spatial smoothing did not enhance the PICSO as significantly as the voxel size effect did. These results accord with those of a previous study, in which the significance of the acquired spatial resolution to the ***σ*/*σ***_**0**_ ratio exceeded that of the smoothing [13].

Consistent with a previous study [13], spatial smoothing greatly enhanced the tSNR, particularly for the data acquired at a high spatial resolution. For example, before spatial smoothing, the average tSNR (±SD) in the PCC increased from 32.8 (±1.9) to 60.5 (±7.4) and to 79.9(±11.2) as the voxel size was increased from 1.3 × 1.3 × 2 to 2 × 2 × 2 mm and to 3 × 3 × 3 mm^3^, respectively. After data sets were spatially smoothed to a fixed FWHM of 5 mm, the average tSNR was 110.3 (±17.0), 115.4 (±22.6), and 105.0 (±19.3) under the aforementioned three acquired voxel sizes, respectively. However, after the data sets were smoothed to a fixed FWHM of 3 mm (Figs 4 and 5), the group-level rsFC with an acquired resolution of 2 × 2 × 2 mm^3^ was significantly higher than that with an acquired resolution of 1.3 × 1.3 × 2 mm^3^ in the voxel-wise paired *t* test (uncorrected p < 0.01), suggesting that the rsFC was more enhanced by increasing the acquired voxel size than by increasing the smoothness. Visual inspection revealed that, when the effective spatial resolution was set at 3 × 3 × 3 mm^3^, as shown in Fig 4, the group-level rsFC with an acquired voxel size of 2 × 2 × 2 mm^3^ was slightly higher than that with an acquired voxel size of 3 × 3 × 3 mm^3^. This could be due to the strategy of smoothing to a predefined FWHM compensating for the confounding factors caused by head motion, which reduced variability among participants and thus enhanced the statistical results [36]. Although the group-level connectivity maps from both acquired voxel sizes appeared to differ, no significant difference was observed in the voxel-wise paired *t* test (uncorrected p < 0.01). Overall, we suggest acquiring data at the desired resolution, rather than smoothing the high-resolution fMRI data to produce reliable rsFC.

## Conclusion

We present the PICSO index as an approximate index for estimating the contributions of physiological fluctuations in spontaneous rs-fMRI oscillations without requiring additional sequence editing or time-consuming ROI selection. In this study, we carefully calibrated the PICSO index by using the phantom data sets at the first step. Second, the resulting PICSO and CS exhibited a high correlation in the thalamic network and DMN. Finally, at a fixed effective spatial resolution, the PICSO values were more enhanced by increasing the acquired voxel size than by increasing the smoothness. These results suggest that, for producing robust rs-fMRI outcomes, directly acquiring functional data at a low spatial resolution is more effective than performing smoothing after acquiring high-resolution data sets. Caution should be exercised when conducting high-resolution acquisition of rs-fMRI signals. In summary, we propose that the PICSO index is an effective sensitivity indicator for rs-fMRI signals, which can be integrated with existing preprocessing procedures to enable quality assurance for future rs-fMRI studies.

## Supporting Information

S1 Table The RETROICOR effect on both *σ*_0_ and *σ* within ten ROIs. (DOCX)
