# Supplementary material for: Physiological Contribution in Spontaneous Oscillations: An Approximate Quality-Assurance Index for Resting-State fMRI Signals
Source: PLoS One. 2016 Feb 12;11(2):e0148393. doi: 10.1371/journal.pone.0148393 (PMC4752279; doi:10.1371/journal.pone.0148393)
Supplement: S1 Table — (DOCX) [file pone.0148393.s001.docx]

**Supporting Information**

**S1 Table. The RETROICOR effect on both** $\boldsymbol{\sigma}_{\boldsymbol{0}}$ **and** $\boldsymbol{\sigma}$ **within ten ROIs**

|  | $\boldsymbol{\sigma}_{\boldsymbol{0}}$ | | $\boldsymbol{\sigma}$ | |  | $\boldsymbol{\sigma}_{\boldsymbol{0}}$ | | $\boldsymbol{\sigma}$ | |
| --- | --- | --- | --- | --- | --- | --- | --- | --- | --- |
| Region of Interest | F | p | F | p | Region of Interest | F | p | F | p |
| L Cerebral Cortex | 0.15 | 0.71 | 15.72 | 0.00* | R Cerebral Cortex | 0.01 | 0.94 | 10.34 | 0.01* |
| L Thalamus | 0.85 | 0.38 | 30.26 | 0.00* | R Thalamus | 0.19 | 0.67 | 20.51 | 0.00* |
| L Caudate | 1.21 | 0.30 | 9.38 | 0.01* | R Caudate | 1.68 | 0.22 | 17.01 | 0.00* |
| L Putamen | 0.21 | 0.65 | 14.36 | 0.00* | R Putamen | 0.00 | 0.99 | 11.59 | 0.01* |
| L Pallidum | 1.63 | 0.23 | 20.33 | 0.00* | R Pallidum | 1.51 | 0.25 | 21.43 | 0.00* |

A p-value of less than 0.05 was considered statistically significant and was designated with one (*) asterisk.

Grey color indicates no significant noise differences in repeated-measure two-way ANOVA tests.
